# Mismatch Negativity of Sad Syllables Is Absent in Patients with Major Depressive Disorder

**DOI:** 10.1371/journal.pone.0091995

**Published:** 2014-03-21

**Authors:** Xiaomei Pang, Jing Xu, Yi Chang, Di Tang, Ya Zheng, Yanhua Liu, Yiming Sun

**Affiliations:** 1 Department of Neurology and Psychiatry, First Affiliated Hospital, Dalian Medical University, Liaoning Province, China; 2 Research Institute of Integrated Traditional and Western Medicine, Dalian Medical University, Liaoning Province, China; 3 Department of Psychology, Dalian Medical University, Liaoning Province, China; University of Jyväskylä, Finland

## Abstract

**Background:**

Major depressive disorder (MDD) is an important and highly prevalent mental disorder characterized by anhedonia and a lack of interest in everyday activities. Additionally, patients with MDD appear to have deficits in various cognitive abilities. Although a number of studies investigating the central auditory processing of low-level sound features in patients with MDD have demonstrated that this population exhibits impairments in automatic processing, the influence of emotional voice processing has yet to be addressed. To explore the automatic processing of emotional prosodies in patients with MDD, we analyzed the ability to detect automatic changes using event-related potentials (ERPs).

**Method:**

This study included 18 patients with MDD and 22 age- and sex-matched healthy controls. Subjects were instructed to watch a silent movie but to ignore the afferent acoustic emotional prosodies presented to both ears while continuous electroencephalographic activity was synchronously recorded. Prosodies included meaningless syllables, such as “dada” spoken with happy, angry, sad, or neutral tones. The mean amplitudes of the ERPs elicited by emotional stimuli and the peak latency of the emotional differential waveforms were analyzed.

**Results:**

The sad MMN was absent in patients with MDD, whereas the happy and angry MMN components were similar across groups. The abnormal sad emotional MMN component was not significantly correlated with the HRSD-17 and HAMA scores, respectively.

**Conclusion:**

The data indicate that patients with MDD are impaired in their ability to automatically process sad prosody, whereas their ability to process happy and angry prosodies remains normal. The dysfunctional sad emotion-related MMN in patients with MDD were not correlated with depression symptoms. The blunted MMN of sad prosodies could be considered a trait of MDD.

## Introduction

Major depressive disorder (MDD) is a highly prevalent mental disorder characterized by anhedonia and reduced interest in activities. Patients with MDD show increased use of medical services, gradual impairment of social and work life, and eventually, overall reduced quality of life.

Patients with MDD also experience deficits in various kinds of cognitive abilities. Converging evidence shows that MDD patients exhibit reduced executive function [1], [2], [3], [4], [5], abnormal psychomotor speed [6], a decrement in vigilance [7], impaired memory [8], [9], [10], [11], [12], [13], and attention deficits [14], [15].

Dysfunction in emotional information processing among MDD patients has been extensively reported in previous studies. Behavioral studies have shown abnormal processing of emotional facial expressions in MDD (reviewed in [16]). For example, one study [17] reported that depressed individuals recognized neutral faces less accurately and more slowly than either happy or sad expressions, although they were equally accurate at recognizing happy and sad faces compared with controls. In a dot-probe study, Gotlib et al. [18] presented emotional faces for 1 second as stimuli and found that depressed participants exhibited attentional bias specifically for sad faces but not for angry or happy faces. However, there was no difference between depressed individuals and healthy controls in a recognition memory test for emotional facial expressions [19]. Functional neuroimaging studies have also described neurobiologically abnormal correlates of depression in a common face-processing network. Some researchers reported increased amygdala activation in response to negative facial expressions such as sad and fearful faces (review in [20]). Recently, event-related potentials (ERPs) have been examined widely in neuroscience studies. Using a task involving the evaluation of emotional intensity, Dai et al. [21] reported larger P1 and P2 components for sad compared with those for other types of faces in patients with MDD, which suggests that those with MDD were more responsive to sad stimuli. Negative attentional bias in MDD was thus demonstrated. Additionally, reduced slow-wave (SW) activity was found in MDD patients, indicating blunted sustained brain activity during processing of positive emotional stimuli [13].

Mismatch negativity (MMN), one cognitive element of ERP, can provide a way to assess automatic neural responses to sensory deviance (MMN; reviewed in [22]). The passive oddball paradigm can be used to assess MMN when the ERPs elicited by standard events are subtracted from the ERPs elicited by deviant events. MMN is generally believed to reflect automatic processing in the temporal and frontal lobes. However, a detailed model of the brain mechanism underlying MMN remains under debate [23]. Over the years, five major hypotheses have been formulated: (a) the change detection hypothesis [24], (b) model adjustment hypothesis [25], (c) adaptation hypothesis [26], (d) novelty detection hypothesis [27], [28], and (e) the prediction error hypothesis [29]. Notably, predictive coding models of MMN have been recently examined (reviewed in [29]). MMN can be thought of as a “prediction error” signal, mismatching the memory-based expectations, which then alerts people to potentially relevant changes or salient information in their surroundings.

MMN has been widely investigated in MDD patients, although the results are not consistent. For example, Kahkonen and colleagues [30] examined the auditory modality and proposed that the MMN amplitude in response to 10% frequency deviance was increased in patients with MDD. He et al. [31] also reported enhanced frequency-MMN amplitudes in treatment-resistant depressed individuals compared with healthy volunteers. In contrast, a recent study conducted by Qiao et al. [32] found that patients with MDD exhibited decreased duration-MMN amplitudes over the frontal–central area under the increment condition (150 ms MMN), although no significant difference between patients and healthy controls was found for temporal MMN regardless of whether the increment or decrement (50 ms MMN) condition was used. They also reported that the MMN of patients with MDD had longer peak latency than did that of healthy controls. Additionally, Takei and colleagues [33] found that the magnetic global field power of pure-tone frequency and the duration MMNm, a magnetic counterpart of MMN, was significantly decreased in those with MDD compared with healthy volunteers. Although these researchers reported dysfunction in the automatic processing of all observed patients with MDD, Umbricht et al. [34] found that both frequency and duration deviance-elicited MMN were normal in MDD. Additionally, Lepisto et al. [35] employed a syllable deviance task and also found unchanged MMN amplitude but shorter MMN latency in children with MDD. Most patients in those studies were in treatment, and evidence has shown that drug therapies such as antidepressants may modify information processing in MDD patients [36]. In view of these findings, it can be argued that basic auditory features such as frequency, duration, and intensity play important roles in automatic processing in MDD patients.

In a study using emotional stimuli, Chang et al. [37] reported dysfunction in processing task-irrelevant emotional faces in patients with MDD patients as reflected by expression-related visual MMN. Their findings revealed that early MMN was smaller and late MMN was absent in MDD patients compared with healthy controls regardless of whether facial expressions were negative or positive.

Emotional tone of voice is essential for verbal communication and social interactions. The ability to perceive emotions from a spoken utterance can be considered an insight into other human beings’ minds including their intentions, attitudes, and feelings [38]. A sudden change in prosody can be a significant indicator of changes in the speaker’s emotions; this marker has the potential to influence the cognition and behavior of others. Some evidence suggests that nonverbal vocal expression of emotion may occur in the early stages of information processing and successfully elicit MMN [39], [40]. For example, Schirmer et al. [39] found that deviant stimuli elicited MMN as an indicator of pre-attentive acoustic change detection. Moreover, Thonnessen et al. [40] reported in an MEG study that, independent of particular acoustic features, MMN can be elicited by changes in emotional prosody. They found early responses (<200 ms) to emotional changes in the bilateral auditory cortices, with more negative amplitude in the right hemisphere.

In the present study, the deviant–standard reverse oddball paradigm [41] was applied to investigate automatic processing of emotional prosody among MDD patients. This paradigm has been shown to minimize the influence of low-level variations in physical features [42]. Given that visual expression-induced MMN is reduced in MDD [37], we hypothesized that MMN elicited by emotional prosody would also be reduced independent of emotional categories.

## Materials and Methods

### Ethics Statement

This study was approved by the Ethics Committee of Dalian Medical University in accordance with Declaration of Helsinki. All participants provided written informed consent to participate in the study after a detailed explanation of the entire procedure.

### Participants

Eighteen patients with MDD recruited from outpatient and inpatient departments at the First Affiliated Hospital of Dalian Medical University in Liaoning, China, were tested. They were matched for gender and age with 22 healthy controls (HC). Consensus diagnoses according to DSM-IV criteria were performed by two trained raters who independently assessed patients and controls using a clinical review. All participants were screened using the Structured Clinical Interview for the Diagnostic and Statistical Manual of Mental Disorders, 4th edition, Patient Edition (SCID) [43]. Severity of depression was evaluated using a Chinese version of the 17-item Hamilton Rating Scale of Depression (HRSD-17); severity of co-morbid anxiety was measured using a Chinese version of the 14-item Hamilton Anxiety Rating Scale (HAMA); cognitive impairment was screened using the Mini-Mental State Examination (MMSE). Group characteristics are shown in Table 1.

**Table 1:** Mean values (with *SD*) of demographic and clinical variables in MDD patients and healthy controls.

	HC(n = 22)	MDD(n = 18)	t/x^2^	*P* Value	*Cohen’s d*
Mean Age(year)	35.55(12.55)	41.00(13.26)	−1.33	0.19	0.42
Sex (Male/Female)	9/13	7/11	0.02	0.90	N/A
HRSD-17	3.00(1.51)	22.44(4.97)	−17.43	<.01	5.29
HAMA	4.36(2.38)	22.17(6.71)	−11.62	<.01	3.54
MMSE	29.86(0.35)	29.56(0.71)	1.80	0.08	0.54
Duration of illness(month)	N/A	13.56(17.77)	N/A	N/A	N/A

HRSD-17, Hamilton Rating Scale of Depression-17; HAMA, Hamilton Anxiety Rating Scale;

MMSE, Mini-Mental State Examination.

Exclusion criteria for all subjects included clear abnormality on brain imaging, a history of major psychiatric disorders other than MDD, major physical illnesses, traumatic brain injury, and taking medications known to affect the central nervous system. Additionally, both the MDD and HC groups reported normal auditory and normal or corrected-to-normal visual acuity. All patients were free of antidepressants, anxiolytics, mood stabilizers, antipsychotics, psychotherapy, and hypnotics for at least 4 weeks at recruitment.

### Stimuli and Procedure

The syllables “dada” spoken with happy, angry, sad and neutral emotional prosodies were produced by female speakers in the same way as in the previous study [39]. Emotional and neutral prosodies were equally long (happy/neutral: 573 ms; angry/neutral: 557 ms; sad/neutral: 557 ms) and loud (happy/neutral: 72 dB max, 64 dB mean; angry/neutral: 67 dB max, 56 dB mean; sad/neutral: 71 dB max, 65 dB mean). The deviant–standard reverse oddball paradigm was used in the present study to eliminate the effect of physical character on emotional prosodies. Under each emotion condition in a given block, the emotional prosodies served as deviants, and the neutral prosodies served as standards. Under each emotion condition in the subsequent block, the neutral prosodies served as deviants, whereas the emotionally expressive prosodies served as standards. Differences in emotional responses were investigated by comparing responses to physically identical stimuli (i.e., the same emotional syllables as both standards and deviants). Participants were presented with six blocks that included 504 standards (*p* = 0.875) and 72 deviants (*p* = 0.125) per block. A minimum of three and a maximum of 11 standards were presented between deviants. The order of blocks was counterbalanced across participants. The stimulus onset asynchrony (SOA) in the present study was 1200 ms.

During the experiment, patients were seated in a comfortable chair in a sound-attenuated and electrically shielded room. The auditory stimuli were delivered over headphones, and the sound volume was set at a comfortable listening level. Patients were asked to ignore the prosody and watch a silent movie shown on a computer monitor 0.8 m in front of the chair. EEG data were recorded when the stimulus sequence was present. Electrode impedance was maintained below 5 kΩ throughout the experiment.

### Data Acquisition and Analysis

Nose-referenced electroencephalogram (EEG) (amplified by SynAmps 2 at bandpass 0.1–100 Hz, sampling rate 500 Hz) was continuously recorded using silver/silver chloride electrodes from 64 electrodes mounted in an elastic cap according to the modified 10–20 system. The electro-oculogram (EOG) was recorded from four electrodes attached above and below the left eye and at the outer canthi of the eyes. Additionally, two recording electrodes were placed at the left and right mastoids. EEG recordings were segmented into epochs of 600 ms, including a prestimulus baseline of 100 ms. Each epoch was averaged offline separately for each stimulus class. The trials with artifacts greater than ±100 μV, which reflected contamination by drifts or muscle movements, were rejected prior to averaging. The mean ERPs were digitally filtered with a low-pass filter at 30 Hz (24 dB/oct). Additionally, the typical artifacts (i.e., horizontal and vertical eye movements and eye blink components) were removed. In the MDD group, the mean number (*SD*) of accepted trials for happy, angry, and sad deviants and happy, angry, and sad standards were 63 (7.61), 62 (11.15), 61 (11.54), 434 (62.05), 433 (68.82), and 440 (53.96), respectively. In the HC group, the mean number (*SD*) of accepted trials for happy, angry, and sad deviants and happy, angry, and sad standards were 61 (11.03), 60 (9.35), 60 (9.86), 413 (78.64), 420 (66.86), and 434 (72.58), respectively. Irrespective of stimulus type (deviant or standard), we found no significant difference in the number of accepted trials either within or between the two groups under the three emotion conditions (*ps* >0.05).

The emotional MMN was calculated by subtracting the ERPs elicited by the emotional syllables serving as standards from the ERPs elicited by the physically identical syllables serving as deviants; this was performed separately for the three emotions (i.e., deviant and standard responses to physically identical emotion stimuli were compared: deviant happy vs. standard happy, deviant angry vs. standard angry, and deviant sad vs. standard sad). MMN topography was explored using electrodes F3, Fz, F4, C3, Cz, C4, P3, Pz, and P4, which provided data on region (frontal/central/parietal) and laterality (left/middle/right) based on scalp position. The waves revealed that emotional MMNs peaked between 130 and 330 ms following stimulus onset for all electrodes, conditions, and participants. The overall MMN peaked at 237 ms (*SD* = 27 ms) following stimulus onset. Individual ERP and MMN amplitudes were calculated as the mean voltage during the 162-ms period centered at the peak latency of the grand average waveform (range: 156–318 ms, mean ±3 *SD*). To examine whether emotional deviants elicited significant MMN, under each condition (happy/angry/sad), the mean amplitude of ERPs was subjected to a repeated-measures analysis of variance (ANOVA) with stimulus type (emotion as deviant vs. emotion as standard), region (frontal vs. central vs. parietal), and lateralization (left vs. middle vs. right) as repeated-measures factors and group (MDD vs. HC) as a between-subjects factor. Under each condition (happy/angry/sad), the peak latency of differential waveforms was subjected to a repeated-measures analysis of variance (ANOVA) with region (frontal vs. central vs. parietal) and lateralization (left vs. middle vs. right) as repeated-measures factors, group (MDD vs. HC) as a between-subjects factor, and age as a covariate.

When necessary, we used the Greenhouse–Geisser adjustment to correct degrees of freedom for nonsphericity. We then used the Bonferroni procedure to conduct post hoc analyses. Effect size was indicated by partial *η*2 value. Pearson correlations were performed between emotional MMN and HRSD-17 and HAMA scores. Data were expressed as mean ± *SD*.

## Results

We found no significant differences between the two groups in age, sex, or MMSE scores. There was a significant difference between MDD patients and healthy participants in scores on the psychometric scales. (See Table 1).

### MMN Amplitude

**Figure 1:**
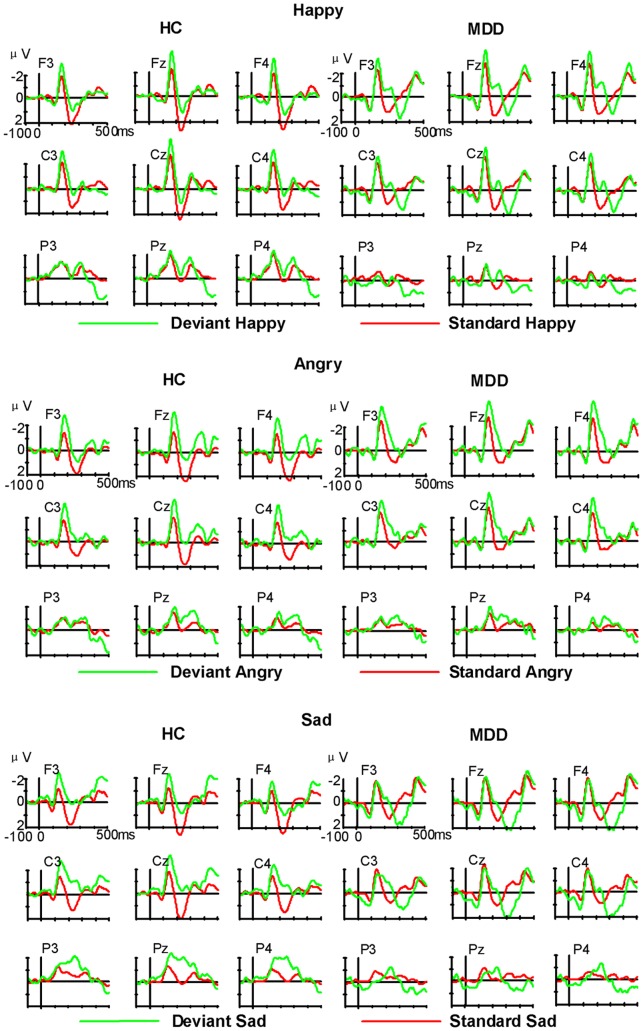
Grand averaged waveforms of ERPs in HC and MDD groups. ERPs elicited by deviant emotional syllables and physically identical standard ones under three conditions.

**Figure 2:**
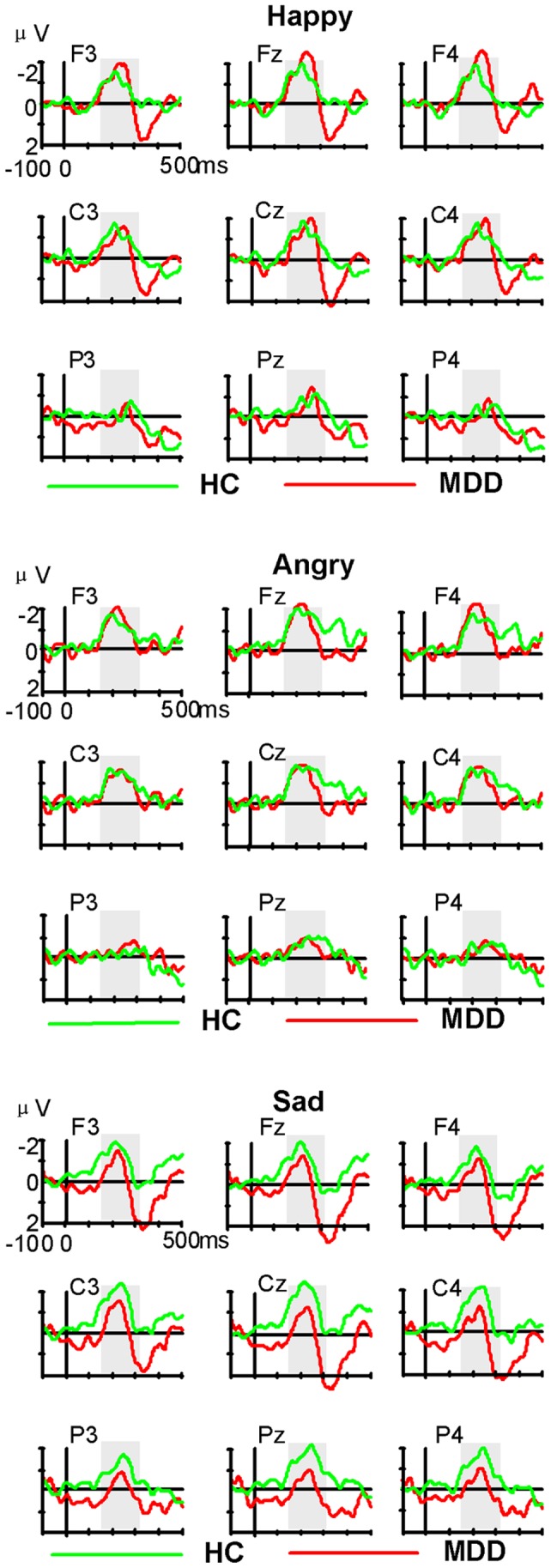
Grand averaged waveforms of differential ERPs (deviant emotion minus standard emotion). Emotional MMN under happy, angry, and sad conditions in HC and MDD groups.

**Figure 3:**
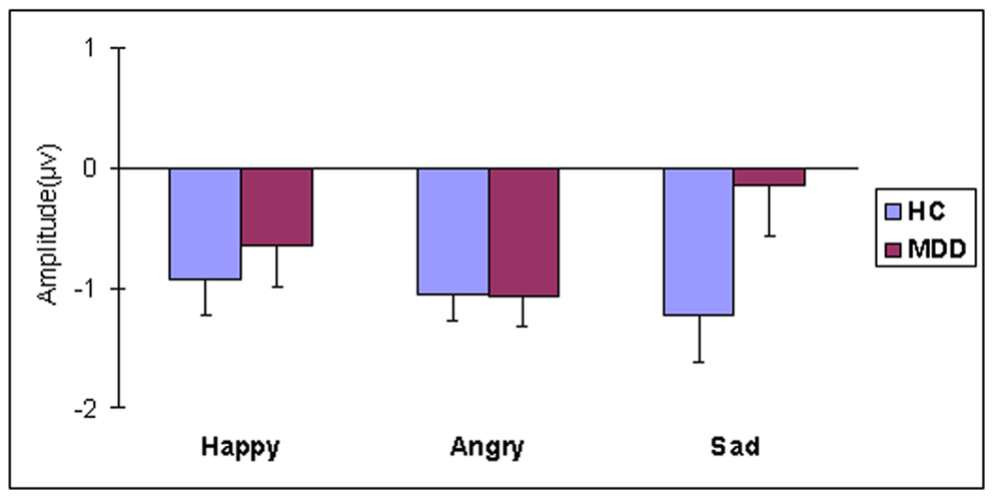
Grand averaged mean amplitudes of MMN. Emotional MMN under happy, angry, and sad conditions in HC and MDD groups. Error bars indicate standard deviations from the mean.

**Figure 4:**
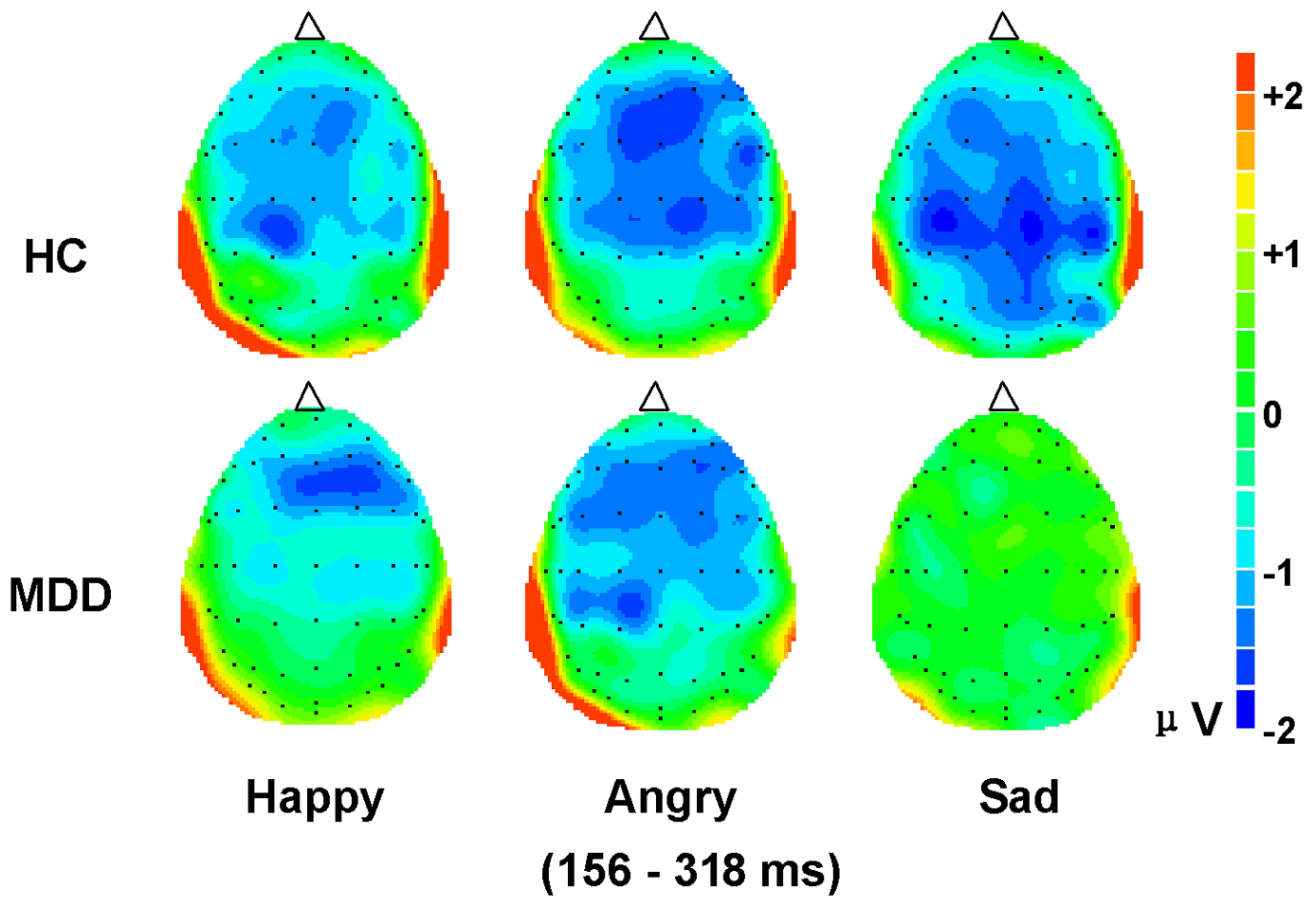
Grand averaged 2D scalp topographies of emotional MMN in HC and MDD groups.

The ERPs responses to standard and deviant emotional stimuli are illustrated in Figure 1. Visual inspection of the difference in waveforms (Figs. 1, 2) indicated the emergence of MMN responses to the three deviant emotional syllables in the 156-318-ms intervals. Figure 3 illustrates the means and SD for the main effect of emotion type and Figure 4 shows the grand averaged topographies of emotional MMN in the HC and MDD groups.

Under the happy condition, we observed a significant main effect of stimulus type (*F*(1, 38) = 11.79, *p*<0.01, partial *η* ^2^ = 0.24), which was due to the more negative responses to deviant happy than to standard happy prosodies. A significant interaction effect of stimulus type×region (*F* (2, 76) = 16.90, *p*<0.01, partial *η* ^2^ = 0.31) was also found, which was due to the more negative response to deviant happy than to standard happy stimuli in the frontal (*p*<0.01) and central (*p*<0.01) regions. A significant interaction effect of stimulus type×lateralization (*F* (2, 76) = 4.00, *p* = 0.02, partial *η* ^2^ = 0.10) reflected a more negative response to deviant happy compared with standard happy stimuli in each lateralization (*ps* <0.05). A significant interaction effect of group×region (*F* (2, 76) = 17.77, *p*<0.01, partial *η* ^2^ = 0.32) was also observed due to the increased amplitudes in the frontal region (*p* = 0.03) and the decreased amplitudes in the parietal region (*p* = 0.02) in the MDD compared with the HC group. Notably, group×stimulus type interaction was not significant (*F*(1, 38) = 0.06, *p* = 0.82, partial *η* ^2^<0.01), indicating that the ERP amplitudes to the happy deviants relative to the amplitudes of happy standards in the MDD and HC groups were equal.

Under the angry condition, a significant main effect of stimulus type (*F*(1, 38) = 45.68, *p*<0.01, partial *η* ^2^ = 0.55) was found, which was due to the more negative response to deviant angry than to standard angry prosodies. A significant interaction effect of stimulus type×region (*F*(2, 76) = 25.32, *p*<0.01, partial *η* ^2^ = 0.40) reflected the more negative response to deviant angry compared with standard angry prosodies in all regions (*ps* <0.05). A significant interaction effect of stimulus type×lateralization (*F* (2, 76) = 6.61, *p*<0.01, partial *η* ^2^ = 0.15) was also observed, which was due to the more negative response to deviant angry than to standard angry prosodies in each lateralization (*ps* <0.05). A significant interaction effect of group×region (*F* (2, 76) = 5.28, *p* = 0.01, partial *η* ^2^ = 0.12) showed increased amplitudes in the frontal region in the MDD group compared with the HC group (*p* = 0.02). Another significant interaction effect of group×region×lateralization (*F*(4, 152) = 3.04, *p* = 0.02, partial *η* ^2^ = 0.07) showed increased amplitudes in the MDD group compared with HC group in the frontal region in each lateralization (*ps* <0.05). Notably, group×stimulus type interaction in angry condition was not significant (*F*(1, 38) = 0.04, *p = *0.84, partial *η* ^2^<0.01), indicating that the ERP amplitudes to the angry deviants relative to the amplitudes of angry standards in the MDD and HC groups were also equal.

Under the sad condition, a significant interaction effect of group×stimulus type (*F*(1, 38) = 4.17, *p*<0.05, partial *η* ^2^ = 0.10) was observed. *Post hoc* analysis revealed a more negative response to deviant sad compared with standard sad stimuli in the HC group (*F*(1, 38) = 10.92, *p*<0.01, partial *η* ^2^ = 0.22), whereas analysis of the amplitudes of the different types of stimuli did not reveal significant effect in the MDD group (*F*(1, 38) = 0.06, *p* = 0.82, partial *η* ^2^<0.01). A significant main effect of lateralization was also observed (*F* (2, 76) = 8.59, *p*<0.01, partial *η* ^2^ = 0.18), as was a significant main effect of region (*F* (2, 76) = 33.78, *p*<0.01, partial *η* ^2^ = 0.47). A stimulus type×region×lateralization interaction (*F*(4, 152) = 6.69, *p*<0.01, partial *η* ^2^ = 0.15) due to the more negative response to deviant sad than that to standard sad stimuli in the left–central (*p*<0.01) and parietal regions (left-parietal: *p* = 0.09, middle-parietal: *p* = 0.02, right-parietal: *p* = 0.02) and a more positive response to standard sad stimuli than that to deviant sad stimuli in the left-middle frontal (*ps* <0.05) and right-middle central (right-central: *p* = 0.06, middle-central: *p* = 0.03) regions was also observed.

### Peak Latency of Differential Waveforms

Under the happy condition, the covariant effect of age was significant (*F*(1, 37) = 9.20, *p*<0.01, partial *η* ^2^ = 0.20), but the main effect of group was not significant, (*F*(1, 37) = 0.09, *p* = 0.77, partial *η* ^2^<0.01). No other main effects or interactions were found.

Under the angry condition, the covariant effect of age was not significant (*F*(1, 37) = 0.01, *p* = 0.91, partial *η* ^2^<0.01), as the main effect of group was also not significant (*F*(1, 37) = 0.06, *p* = 0.82, partial *η* ^2^<0.01). The main effect of region was significant (*F* (2, 74) = 5.07, *p* = 0.01, partial *η* ^2^ = 0.12) due to the longer latency in the parietal (245.3±6.6 ms) than in the frontal (220.7±6.2 ms, *P*<0.01) or central (226.1±6.8 ms, *P* = 0.01) region. No other main effects or interactions were found.

Under the sad condition, the covariant effect of age was not significant (*F*(1, 37) = 0.77, *p* = 0.39, partial *η* ^2^ = 0.02). The main effect of group was also nonsignificant (*F*(1, 37) = 0.10, *p* = 0.76, partial *η* ^2^<0.01). No other main effects or interactions were found.

### Correlation Analysis

Among patients with MDD, the amplitudes of happy MMN were marginally significantly correlated with HRSD-17 (*r* = 0.43, *P* = 0.07) and HAMA (*r* = 0.42, *P* = 0.09) scores; the latencies of happy MMN were not significantly correlated with HRSD-17 (r = −0.25, *P* = 0.33) or HAMA *(r* = −0.29, P = 0.24) scores.

The amplitudes and latencies of angry MMN in patients with MDD were not significantly correlated with HRSD-17 (*r* = 0.12, *P* = 0.65; *r* = −0.14, *P* = 0.57, respectively) or HAMA (*r* = 0.06, P = 0.83; *r* = 0.03, *P* = 0.91, respectively) scores.

The amplitudes and latencies of sad MMN in patients with MDD were not significantly correlated with HRSD-17 (*r* = −0.09, *P* = 0.73; *r* = 0.38, *P* = 0.12, respectively) or HAMA (*r* = −0.32, *P* = 0.20; *r* = 0.35, *P* = 0.16, respectively) scores.

## Discussion

In this study, we investigated automatic processing of auditory emotional stimuli in MDD patients and healthy controls. The results revealed the following: (1) sad MMN was absent in patients with MDD; (2) happy and angry MMN were similar across groups; (3) the blunted sad emotion-based MMN was not correlated with the severity of depressive symptoms as reflected in HRSD-17 scores.

As described in the Introduction, only one study of emotional MMN in MDD patients has been reported. Chang et al. [37] investigated task-irrelevant processing of facial expressions in MDD patients by recording expression-related MMN. They found that when processing task-irrelevant emotional faces, early MMN in the MDD group was reduced, and late MMN was absent compared with healthy controls independent of whether the facial expressions were negative or positive. The results of Chang et al.’s study [37] are partially in line with our finding that sad MMN was absent in patients with MDD compared with the healthy controls. Notably, in Chang et al.’s study, ambiguous emotionally negative schematic faces were used, which could be interpreted as sad or angry. Taken together, it is likely reasonable that the abnormality in automatic emotional processing in MDD arose from sad emotion more than angry emotion. However, the MMN elicited by happy prosody did not differ between patients with MDD and healthy controls, which is inconsistent with the findings reported by Chang et al. [37]. This difference in results may arise from variability in the sensory modalities investigated in these two studies.

Result also indicated the change induced by auditory sad emotion-based MMN was unrelated to the severity of depressive symptoms as reflected in HRSD-17 scores. This pattern was also found in visual modality studies [37], [44]. These findings provide evidence that cognitive impairment is persistent even after MDD symptoms have been alleviated or HRSD-17 scores are reduced [45], [46]. Therefore, it is reasonable that the dysfunctional automatic change detection reflected by MMN may be unrelated to depressive symptoms in patients with MDD. In brief, the findings indicate that the blunted MMN of sad prosody can be considered a trait, not a state, abnormality in MDD.

The interaction effect of group by region was found for both happy and angry emotion in the identical stimuli comparison for the ERP amplitudes. It suggested that the processing of both the happy and angry prosodies is frontally enhanced in the MDD group comparing with HC group. Considering anger and happiness are both high arousal emotions [47], but valence varies, this finding may indicate that MDD patients are more sensitive to high arousal stimuli than healthy volunteers. Another explanation may lie in different response to novelty stimuli of MDD patients. However, there is no evidence that anger and happiness are more novel than sadness. Moreover, the lack of group difference in the N2 potential in a MDD novelty processing study [48] makes this explanation implausible.

Before concluding, it is important to note this study’s limitations. First, the study was conducted with a relatively small number of participants. Future studies should try to replicate the findings with a larger sample. Second, the drug washout period lasted for 4 weeks, and one may argue that the prior exposure to psychotropic medications in the depressed sample may have affected the MMN findings. Wienberg et al. [49], [50] reported that both low (10 mg) and high (15 mg) doses of escitalopram administered to healthy volunteers increased frequency-MMN amplitudes. Another two studies [51], [52] found no effect of psychotropic medications on MMN in healthy volunteers. Moreover, Takei et al. [33] reported no significant correlation of pure-tone frequency and duration of MMNm with antidepressants, anxiolytics, and hypnotics in patients with MDD. Taken together, these findings indicate that psychotropic medication does not seem to be responsible for the absent sad MMN in depressed patients. Third, the effect of stage of illness was not addressed in the present study. A study [53] investigated the differences of MMN in patients with first-episode schizophrenia at acute and post-acute phases and found that the MMN amplitude of patients in the acute phase did not differ from that of controls, whereas it was reduced in the post-acute phase. Thus, MMN may be modulated by the MDD patients’ stage of illness. And this should be considered in future studies in depressed patients.

These limitations notwithstanding, this study is, to our knowledge, the first to report automatic responses to happy, angry, and sad prosodies in patients with MDD relative to healthy comparison subjects. We demonstrated a blunted automatic response to sad prosodies in patients with MDD, whereas happy and angry MMN were similar across groups. Furthermore, sad MMN appears to be a trait, rather than state, abnormality.

## Supporting Information

Text S1 **The analysis of mean amplitude values from the 300–400 ms latency.**
(DOC)

## References

[1] Fossati P, Ergis AM, Allilaire JF (2002) Executive functioning in unipolar depression: a review. Encephale 28: 97–107. 11972136

[2] Merriam EP, Thase ME, Haas GL, Keshavan MS, Sweeney JA (1999) Prefrontal cortical dysfunction in depression determined by Wisconsin Card Sorting Test performance. Am J Psychiatry 156: 780–782. 10327916 10.1176/ajp.156.5.780

[3] Paelecke-Habermann Y, Pohl J, Leplow B (2005) Attention and executive functions in remitted major depression patients. J Affect Disord 89: 125–135. 16324752 10.1016/j.jad.2005.09.006

[4] Paradiso S, Lamberty GJ, Garvey MJ, Robinson RG (1997) Cognitive impairment in the euthymic phase of chronic unipolar depression. J Nerv Ment Dis 185: 748–754. 9442186 10.1097/00005053-199712000-00005

[5] Schatzberg AF, Posener JA, DeBattista C, Kalehzan BM, Rothschild AJ, et al (2000) Neuropsychological deficits in psychotic versus nonpsychotic major depression and no mental illness. Am J Psychiatry 157: 1095–1100. 10873917 10.1176/appi.ajp.157.7.1095

[6] Buyukdura JS, McClintock SM, Croarkin PE (2011) Psychomotor retardation in depression: biological underpinnings, measurement, and treatment. Prog Neuropsychopharmacol Biol Psychiatry 35: 395–409. 21044654 10.1016/j.pnpbp.2010.10.019 PMC3646325

[7] Egeland J, Rund BR, Sundet K, Landro NI, Asbjornsen A, et al (2003) Attention profile in schizophrenia compared with depression: differential effects of processing speed, selective attention and vigilance. Acta Psychiatr Scand 108: 276–284. 12956828 10.1034/j.1600-0447.2003.00146.x

[8] Basso MR, Bornstein RA (1999) Relative memory deficits in recurrent versus first-episode major depression on a word-list learning task. Neuropsychology 13: 557–563. 10527064 10.1037//0894-4105.13.4.557

[9] Bearden CE, Glahn DC, Monkul ES, Barrett J, Najt P, et al (2006) Patterns of memory impairment in bipolar disorder and unipolar major depression. Psychiatry Res 142: 139–150. 16631256 10.1016/j.psychres.2005.08.010

[10] Lim SL, Kim JH (2005) Cognitive processing of emotional information in depression, panic, and somatoform disorder. J Abnorm Psychol 114: 50–61. 15709812 10.1037/0021-843X.114.1.50

[11] Pelosi L, Slade T, Blumhardt LD, Sharma VK (2000) Working memory dysfunction in major depression: an event-related potential study. Clin Neurophysiol 111: 1531–1543. 10964062 10.1016/s1388-2457(00)00354-0

[12] Purcell R, Maruff P, Kyrios M, Pantelis C (1997) Neuropsychological function in young patients with unipolar major depression. Psychol Med 27: 1277–1285. 9403899 10.1017/s0033291797005448

[13] Shestyuk AY, Deldin PJ, Brand JE, Deveney CM (2005) Reduced sustained brain activity during processing of positive emotional stimuli in major depression. Biol Psychiatry 57: 1089–1096. 15866547 10.1016/j.biopsych.2005.02.013

[14] El Massioui F, Everett J, Martin MT, Jouvent R, Widlocher D (1996) Attention deficits in depression: an electrophysiological marker. Neuroreport 7: 2483–2486. 8981408 10.1097/00001756-199611040-00016

[15] Weiland-Fiedler P, Erickson K, Waldeck T, Luckenbaugh DA, Pike D, et al (2004) Evidence for continuing neuropsychological impairments in depression. J Affect Disord 82: 253–258. 15488254 10.1016/j.jad.2003.10.009

[16] Bourke C DK, Porter R (2010) Processing of facial emotion expression in major depression: a review. Aust N Z J Psychiatry 44: 681–696. 20636189 10.3109/00048674.2010.496359

[17] Leppänen JM, Milders M, Bell JS, Terriere E, Hietanen JK (2004) Depression biases the recognition of emotionally neutral faces. Psychiatry Res 128: 123–133. 15488955 10.1016/j.psychres.2004.05.020

[18] Gotlib IH, Krasnoperova E, Yue DN, Joormann J (2004) Attentional biases for negative interpersonal stimuli in clinical depression. J Abnorm Psychol 113: 121–135. 14992665 10.1037/0021-843X.113.1.121

[19] Ridout N, Astell A, Reid I, Glen T, O’Carroll R (2003) Memory bias for emotional facial expressions in major depression.Cogn Emot. 17: 101–122. 10.1080/02699930302272 29715743

[20] Stuhrmann A, Suslow T, Dannlowski U (2011) Facial emotion processing in major depression: a systematic review of neuroimaging findings. Biol Mood Anxiety Disord 1: 10. 22738433 10.1186/2045-5380-1-10 PMC3384264

[21] Dai Q, Feng Z (2012) More excited for negative facial expressions in depression: evidence from an event-related potential study. Clin Neurophysiol 123: 2172–2179. 22727714 10.1016/j.clinph.2012.04.018

[22] Paavilainen P (2013) The mismatch-negativity (MMN) component of the auditory event-related potential to violations of abstract regularities: A review. Int J Psychophysiol 88: 109–123. 23542165 10.1016/j.ijpsycho.2013.03.015

[23] Lieder F, Daunizeau J, Garrido MI, Friston KJ, Stephan KE (2013) Modelling trial-by-trial changes in the mismatch negativity. PLoS Comput Biol 9: e1002911. 23436989 10.1371/journal.pcbi.1002911 PMC3578779

[24] Schröger E, Winkler I (1995) Presentation rate and magnitude of stimulus deviance effects on human pre-attentive change detection. Neurosci Lett 193: 185–188. 7478179 10.1016/0304-3940(95)11696-t

[25] Winkler I, Czigler I (1998) Mismatch negativity: deviance detection or the maintenance of the ‘standard’. Neuroreport 9: 3809–3813. 9875709 10.1097/00001756-199812010-00008

[26] May PJ, Tiitinen H (2010) Mismatch negativity (MMN), the deviance-elicited auditory deflection, explained. Psychophysiology 47: 66–122. 19686538 10.1111/j.1469-8986.2009.00856.x

[27] Escera C, Corral M (2007) Role of mismatch negativity and novelty-P3 in involuntary auditory attention. J Psychophysiol 21: 251–264.

[28] Tiitinen H, May P, Reinikainen K, Näätänen R (1994) Attentive novelty detection in humans is governed by pre-attentive sensory memory. Nature 372: 90–92. 7969425 10.1038/372090a0

[29] Winkler I, Czigler I (2012) Evidence from auditory and visual event-related potential (ERP) studies of deviance detection (MMN and vMMN) linking predictive coding theories and perceptual object representations. Int J Psychophysiol 83: 132–143. 22047947 10.1016/j.ijpsycho.2011.10.001

[30] Kahkonen S, Yamashita H, Rytsala H, Suominen K, Ahveninen J, et al (2007) Dysfunction in early auditory processing in major depressive disorder revealed by combined MEG and EEG. J Psychiatry Neurosci 32: 316–322. 17823647 PMC1963351

[31] He W, Chai H, Zheng L, Yu W, Chen W, et al (2010) Mismatch negativity in treatment-resistant depression and borderline personality disorder. Prog Neuropsychopharmacol Biol Psychiatry 34: 366–371. 20074609 10.1016/j.pnpbp.2009.12.021

[32] Qiao Z, Yu Y, Wang L, Yang X, Qiu X, et al (2013) Impaired pre-attentive change detection in major depressive disorder patients revealed by auditory mismatch negativity. Psychiatry Res 211: 78–84. 23149029 10.1016/j.pscychresns.2012.07.006

[33] Takei Y, Kumano S, Hattori S, Uehara T, Kawakubo Y, et al (2009) Preattentive dysfunction in major depression: a magnetoencephalography study using auditory mismatch negativity. Psychophysiology 46: 52–61. 19055502 10.1111/j.1469-8986.2008.00748.x

[34] Umbricht D, Koller R, Schmid L, Skrabo A, Grubel C, et al (2003) How specific are deficits in mismatch negativity generation to schizophrenia? Biol Psychiatry 53: 1120–1131. 12814863 10.1016/s0006-3223(02)01642-6

[35] Lepisto T, Soininen M, Ceponiene R, Almqvist F, Naatanen R, et al (2004) Auditory event-related potential indices of increased distractibility in children with major depression. Clin Neurophysiol 115: 620–627. 15036058 10.1016/j.clinph.2003.10.020

[36] Pringle A, McCabe C, Cowen P, Harmer C (2013) Antidepressant treatment and emotional processing: can we dissociate the roles of serotonin and noradrenaline? J Psychopharmacol 27: 719–731. 23392757 10.1177/0269881112474523

[37] Chang Y, Xu J, Shi N, Zhang B, Zhao L (2010) Dysfunction of processing task-irrelevant emotional faces in major depressive disorder patients revealed by expression-related visual MMN. Neurosci Lett 472: 33–37. 20117175 10.1016/j.neulet.2010.01.050

[38] Bruck C, Kreifelts B, Wildgruber D (2011) Emotional voices in context: a neurobiological model of multimodal affective information processing. Phys Life Rev 8: 383–403. 22035772 10.1016/j.plrev.2011.10.002

[39] Schirmer A, Striano T, Friederici AD (2005) Sex differences in the preattentive processing of vocal emotional expressions. Neuroreport 16: 635–639. 15812323 10.1097/00001756-200504250-00024

[40] Thonnessen H, Boers F, Dammers J, Chen YH, Norra C, et al (2010) Early sensory encoding of affective prosody: neuromagnetic tomography of emotional category changes. Neuroimage 50: 250–259. 19969096 10.1016/j.neuroimage.2009.11.082

[41] Jacobsen T, Schroger E (2003) Measuring duration mismatch negativity. Clin Neurophysiol 114: 1133–1143. 12804682 10.1016/s1388-2457(03)00043-9

[42] Xu Q, Yang Y, Wang P, Sun G, Zhao L (2013) Gender Differences in Preattentive Processing of Facial Expressions: An ERP Study. Brain Topogr 26: 488–500. 23371479 10.1007/s10548-013-0275-0

[43] First M, Spitzer R, Gibbon M, Wiliams J (1995) Structured Clinical Interview for DSM-IV Axis I Disorders: Patient Edition (SCID-I/P) Version 2.0, American Psychiatric Press, Washington, DC.

[44] Qiu X, Yang X, Qiao Z, Wang L, Ning N, et al (2011) Impairment in processing visual information at the pre-attentive stage in patients with a major depressive disorder: a visual mismatch negativity study. Neuroscience Lett 491: 53–57. 10.1016/j.neulet.2011.01.006 21215792

[45] Hammar A (2003) Automatic and effortful information processing in unipolar major depression. Scand J Psychol 44: 409–413. 15030106 10.1046/j.1467-9450.2003.00361.x

[46] Preiss M, Kucerova H, Lukavsky J, Stepankova H, Sos P, et al (2009) Cognitive deficits in the euthymic phase of unipolar depression. Psychiatry Res 169: 235–239. 19765829 10.1016/j.psychres.2008.06.042

[47] Weierich MR, Wright CI, Negreira A, Dickerson BC, Barrett LF (2010) Novelty as a dimension in the affective brain. NeuroImage 49: 2871–2878. 19796697 10.1016/j.neuroimage.2009.09.047 PMC2818231

[48] Bruder GE, Kroppmann CJ, Kayser J, Stewart JW, McGrath PJ, et al (2009) Reduced brain responses to novel sounds in depression: P3 findings in a novelty oddball task. Psychiatry Res 170: 218–223. 19900720 10.1016/j.psychres.2008.10.023 PMC3341094

[49] Oranje B, Jensen K, Wienberg M, Glenthoj BY (2008) Divergent effects of increased serotonergic activity on psychophysiological parameters of human attention. Int J Neuropsychopharmacol 11: 453–463. 17971261 10.1017/S1461145707008176

[50] Wienberg M, Glenthoj BY, Jensen KS, Oranje B (2010) A single high dose of escitalopram increases mismatch negativity without affecting processing negativity or P300 amplitude in healthy volunteers. J Psychopharmacol 24: 1183–1192. 19304862 10.1177/0269881109102606

[51] Umbricht D, Vollenweider FX, Schmid L, Grubel C, Skrabo A, et al (2003) Effects of the 5-HT2A agonist psilocybin on mismatch negativity generation and AX-continuous performance task: implications for the neuropharmacology of cognitive deficits in schizophrenia. Neuropsychopharmacology 28: 170–181. 12496954 10.1038/sj.npp.1300005

[52] Leung S, Croft RJ, Guille V, Scholes K, O’Neill BV, et al (2010) Acute dopamine and/or serotonin depletion does not modulate mismatch negativity (MMN) in healthy human participants. Psychopharmacology 208: 233–244. 20012022 10.1007/s00213-009-1723-0

[53] Devrim-Ucok M, Keskin-Ergen HY, Ucok A (2008) Mismatch negativity at acute and post-acute phases of first-episode schizophrenia. Eur Arch Psychiatry Clin Neurosci 258: 179–185. 18000635 10.1007/s00406-007-0772-9

