# Supplementary material for: Mismatch Negativity of Sad Syllables Is Absent in Patients with Major Depressive Disorder
Source: PLoS One. 2014 Mar 21;9(3):e91995. doi: 10.1371/journal.pone.0091995 (PMC3962367; doi:10.1371/journal.pone.0091995)
Supplement: Text S1 — The analysis of mean amplitude values from the 300–400 ms latency. (DOC) [file pone.0091995.s001.doc]

Visual inspection of the difference in waveforms (Fig. 2) clearly revealed that there is a difference around 300-400 ms latency in response amplitudes. And thus the differential responses around 300-400 ms latency in response amplitudes between two groups were analyzed. Under each condition (happy/angry/sad), the mean amplitude of ERP responses was subjected to a repeated-measures analysis of variance (ANOVA) with stimulus type (emotion as deviant vs. emotion as standard), region (frontal vs. central), and lateralization (left vs. middle vs. right) as repeated-measures factors and group (MDD vs. HC) as a between-subjects factor.

Results

Under the happy condition, we observed a marginally significant interaction effect of group × region (*F* (1, 38) = 3.66, *p* = 0.06, partial *η*^2^ = 0.09), which was due to the more positive response to happy prosody in MDD group than that in HC group in the central region. A significant interaction effect of group × region × lateralization (*F* (2, 76) = 3.75, *p* = 0.03, partial *η*^2^ = 0.09) reflected a more positive response to happy prosody in MDD group than that in HC group in right-central region (*p =* 0.04) and a more negative response to happy prosody in HC group than that in MDD group in middle-central region(*p <* 0.05) .

Under the angry condition, a significant main effect of lateralization (*F* (2, 76) = 4.57, *p* = 0.01, partial *η*^2^ = 0.11) was found, which was due to the more negative response to angry prosody in middle lateralization than that in left (*p* = 0.03) and right one (*p* = 0.02). A significant interaction effect of group × lateralization (*F* (2, 76) = 3.18, *p* < 0.05, partial *η*^2^ = 0.08) reflected the more negative response to angry prosody in middle lateralization than that in left (*p* < 0.01) and right one (*p* = 0.02) in HC group whereas the effect was lack in MDD group (*ps* >0.05). A significant interaction effect of group × stimulus type × lateralization (*F* (2, 76) = 5.10, *p* = 0.01, partial *η*^2^ = 0.12) was also observed, which was due to the more negative response to deviant angry prosody than that to standard stimuli in middle (*p* = 0.01) and right lateralization (*p* = 0.01) in HC group whereas the effect was lack in MDD group (*ps* > 0.05).

Under the sad condition, a significant main effect of stimulus type was observed (*F*(1, 38) = 6.25, *p* = 0.02, partial *η*^2^ = 0.14), and a significant interaction effect of group × stimulus type (*F*(1, 38) = 6.17, *p* = 0.02, partial *η*^2^ = 0.14) was also observed. *Post hoc* analysis revealed a more positive response to deviant sad in the MDD group than that in the HC group (*p* < 0.05); deviant sad prosody elicited more positive response than standard one in MDD group (*p* < 0.01) whereas the analysis of amplitudes of the different types of stimuli did not show significant effect in the HC group (*p* = 0.99). There is also a significant main effect of lateralization (*F* (2, 76) = 7.46, *p* < 0.01, partial *η*^2^ = 0.16) and a significant interaction effect of stimulus type × lateralization (*F* (2, 76) = 4.70, *p* = 0.01, partial *η*^2^ = 0.11) due to a more positive response to deviant sad than to standard sad stimuli in right lateralization (*p =* 0.01) and the more negative response to sad standard than to sad deviant stimuli in left (*p =* 0.05) and middle lateralization (*p =* 0.02) . A marginally significant region × stimulus type interaction (*F* (1, 38) = 3.44, *p* = 0.07, partial *η*^2^ = 0.08) due to a more positive response to deviant sad than that to standard sad stimuli in the frontal region (*p* = 0.01) and a more negative response to standard sad than that to deviant sad stimuli in the central region (*p* = 0.05) was also observed.
